# Understanding parental trauma in caring for children with down syndrome within LSEN school settings

**DOI:** 10.3389/frcha.2026.1926429

**Published:** 2026-10-08

**Authors:** Habeeb Omoponle Adewuyi, Veronica M. Dwarika

**Affiliations:** Department of Educational Psychology, University of Johannesburg, Johannesburg, South Africa

**Keywords:** caregiving stress, Down syndrome, LSEN schools, parental trauma, post-traumatic growth, systemic betrayal

## Abstract

**Background:**

Parents raising children with Down syndrome (DS) encounter multifaceted adversities that may precipitate trauma responses, yet the intersection of caregiving, systemic barriers, and trauma remains underexplored in low-resourced educational contexts.

**Aim:**

This study examined the nature, sources, and manifestations of parental trauma among caregivers of children with DS enrolled in Learners with Special Educational Needs (LSEN) schools in Johannesburg, South Africa.

**Method:**

Using an interpretative phenomenological design, semi-structured interviews and a focus group were conducted with eight purposively selected parents (seven mothers, one father) whose children attended an LSEN school in Johannesburg. Data were analysed following Interpretative Phenomenological Analysis procedures.

**Findings:**

Four superordinate themes emerged: (1) traumatic shock at diagnosis and systemic betrayal; (2) cumulative caregiving strain as chronic trauma exposure; (3) secondary trauma from institutional gatekeeping and stigma; and (4) precarious resilience and post-traumatic growth. Parents described diagnostic experiences marked by insensitive communication, fragmented healthcare pathways, and educational exclusion that functioned as compound traumatic events. Chronic hypervigilance, anticipatory grief, and moral injury from inadequate institutional support constituted sustained trauma burdens.

**Conclusion:**

Parental trauma in LSEN contexts arises from the convergence of individual psychological responses, systemic failures, and structural violence.

## 1 Introduction

The transition to parenthood, under typical circumstances, involves adaptation, joy, and normative stress. When a child receives a diagnosis of Down syndrome (DS), however, the psychological landscape shifts dramatically. For many parents, the diagnostic moment becomes what psychological literature terms a “potentially traumatic event”, an encounter with threat, loss, or profound disruption that overwhelms ordinary coping capacities (1). A potentially traumatic event does not automatically become trauma; it is the presence or absence of protective relational and institutional responses at the moment of diagnosis, and afterwards, that determines whether the encounter is integrated without lasting harm or becomes a source of sustained psychological injury (2). Yet the potential trauma of parenting a child with DS does not necessarily end at the consultation room door. Rather, for caregivers navigating under-resourced educational and healthcare systems, trauma may become a chronic condition, renewed and deepened by each encounter with institutional indifference, each rejection from a mainstream school, and each moment of public shaming (3, 4).

The concept of parental trauma in the context of childhood disability remains theoretically underdeveloped and empirically underexamined, particularly in low- and middle-income countries. Much of the existing literature has focused on parental stress, burden, or depression, constructs that capture distress but fail to capture the specific phenomenology of trauma, including intrusions, hyperarousal, avoidance, and disruptions to meaning-making systems (4, 5). It is important to state this precisely: it is not Down syndrome itself, but the low-resourced and often unsupportive context surrounding diagnosis and caregiving, that carries the potentially traumatic features described in this literature (see also work on communicating a Down syndrome diagnosis, and the broader literature on stigma toward people with intellectual disabilities). Trauma, unlike general stress, involves a fundamental shattering of assumptions about safety, justice, and predictability (2). For parents of children with DS, these assumptions may be shattered not once but repeatedly: when a healthcare provider delivers a diagnosis without compassion, when a school principal refuses admission at the gate, when a community member stares or whispers, or when a child experiences a medical crisis that the system is ill-equipped to manage.

South Africa presents a particularly instructive context for examining parental trauma. The country's constitutional commitment to disability rights and inclusive education, articulated in the White Paper 6 (6) and the Screening, Identification, Assessment and Support (SIAS) policy (7), stands in stark tension with the lived realities of families navigating fragmented services, transport poverty, and persistent stigma (8, 9). This policy-practice gap may itself be a source of trauma, what some scholars term “systemic betrayal”, wherein the institutions designed to protect and support families become sources of harm through neglect, delay, or outright exclusion (10). In the South African context, this can include a referral hospital failing to fast-track a newborn with suspected DS for cardiac screening, a school administrator turning a family away at the gate without following the legally required admission process, or a social welfare office allowing a disability grant to lapse without notice; each is an ordinary institutional function that, when withheld, becomes a source of harm rather than protection.

Learners with Special Educational Needs (LSEN) schools occupy an ambiguous position within this landscape. These specialised institutions serve as essential havens for many children with DS, providing adapted curricula, on-site therapies, and smaller class sizes (11, 12). LSEN schools operate within South Africa's inclusive education policy architecture, set out in White Paper 6 (6) and the SIAS policy (7), which envisages a continuum of provision in which LSEN placement is one option alongside full inclusion in mainstream schools rather than a substitute for it. The present study does not treat LSEN placement as inherently preferable to mainstream inclusion; rather, it treats LSEN enrolment, for these families, as a context that followed prior exclusion from mainstream settings, and asks how that context both mitigates and reproduces the traumatic and structural pressures under study. Moreover, LSEN schools, despite their strengths, operate in severely resource-constrained environments, with limited budgets, high staff-to-learner ratios, and minimal mental health support for caregivers (13). Thus, while LSEN schools may mitigate some sources of trauma, they cannot, in isolation, address the structural conditions that generate traumatic stress in the first place.

Against this backdrop, the current study explores the lived experiences of trauma among parents who are raising children with Down syndrome and who are enrolled in LSEN schools in Johannesburg. More precisely, it examines the nature and manifestations of parental trauma, including the emotional and practical dimensions, and explores how systemic barriers, institutional responses, and everyday interpersonal interactions shape parental experience. It also attends to the support and resources that facilitate parents’ coping, resilience, and post-traumatic growth. In this study, “everyday interpersonal interactions” refers to parents’ direct encounters with healthcare workers, school staff, and community members in the course of ordinary caregiving and school life, and “parental experience” refers to parents’ own first-person accounts of the emotional, practical, and relational dimensions of raising a child with DS, as elicited through interview and focus group data. Using an interpretative phenomenological approach, the study aims to develop insights grounded in the local context and to contribute to the wider literature on disability, caregiving, and trauma in the Global South. Ultimately, the findings are expected to guide the crafting of trauma-informed policies and practices to strengthen the support systems for families with children who have Down syndrome.

## 2 Theoretical framework

This study is guided by Ungar's (14) social-ecological model of resilience and by trauma theory, specifically the concept of systemic betrayal (15). Ungar's framework conceptualises resilience not as an individual trait but as an emergent property of interactions among people, their environments, families, schools, communities, and policy systems. Trauma, similarly, is not merely an intrapsychic event but is shaped by the availability (or absence) of responsive, trustworthy institutional support. The concept of betrayal trauma extends this insight by arguing that harm inflicted by trusted or depended-upon systems (healthcare, education, welfare) is particularly damaging because it undermines the very foundations of safety and trust (15).

The integration of these frameworks allows for a nuanced analysis of parental trauma in LSEN contexts. Parents of children with DS depend on schools, hospitals, and social services not as optional extras but as essential infrastructure for daily survival and child wellbeing. When these systems fail, through diagnostic insensitivity, placement delays, transport breakdowns, or communication failures, the harm is not merely instrumental but existential. Diagnostic insensitivity, as used here, refers to how a DS diagnosis is communicated and followed up: the absence of preparation, privacy, plain-language explanation, or timely referral to psychosocial and medical support, leaving parents to interpret the diagnosis largely on their own. Parents may experience not only frustration but a profound sense of betrayal, which may manifest as hypervigilance, avoidance of institutional engagement, and disruptions in meaning-making (16, 17). This shift from frustration to betrayal is not simply a matter of degree: frustration is a response to a single unmet expectation, whereas betrayal reflects the parent's revised understanding that an institution they depended on for their child's safety and wellbeing cannot, in fact, be relied upon, a reappraisal that alters how future encounters with that institution are anticipated and experienced (15).

Furthermore, the social-ecological perspective highlights that trauma responses are not uniformly distributed. Parents’ material resources, social networks, cultural frameworks, and prior histories shape both exposure to potentially traumatic events and the availability of protective factors. In the South African context, where socio-economic inequality is extreme, and cultural beliefs about disability vary widely, these differential exposures and resources are particularly consequential (10). To be explicit, it is not the presence of Down syndrome in the family that this study treats as the source of trauma, but the absence of adequate institutional and relational support around it. This study adopts the American Association on Intellectual and Developmental Disabilities’ (AAIDD) twelfth-edition definition of intellectual disability, which conceptualises intellectual disability in terms of support needs rather than deficits, and situates functioning as the product of the interaction between the person and their environment (18). Read through this lens, and consistent with the interdependence and relational autonomy that this framework foregrounds, disability is not itself pathogenic; what generates traumatic stress is the mismatch between a child's support needs and the support that under-resourced systems can provide. This study, therefore, attends to the intersection of structural, relational, and individual dimensions of trauma.

Building on this AAIDD-consistent, supports-based understanding of intellectual disability, this study establishes that parental trauma in raising a child with Down syndrome within LSEN settings is a systemic phenomenon, not merely an individual psychological response, and not a phenomenon produced by the child's disability in itself. It arises from diagnostic experiences, chronic caregiving strain, institutional gatekeeping, and community stigma, creating a dynamic traumatic ecology. Addressing it requires institutional and structural interventions, not just therapeutic support.

Three interconnected domains shape traumatic exposure. First, Diagnostic and Healthcare Pathways—insensitive communication, fragmented appointments, and lack of psychosocial support—initiate and compound trauma. Second, Institutional Gatekeeping and Educational Access—school rejection, stigma, and inadequate resourcing—generate secondary trauma through systemic betrayal. Third, Caregiving Precarity and Resource Scarcity—transport poverty, grant inadequacy, chronic medical surveillance, and anticipatory grief—constitute sustained complex trauma that erodes adaptive coping. Trauma theory has previously been applied to parents of children with intellectual disabilities primarily through the study of parental post-traumatic stress symptoms and their association with caregiving burden (5, 17); the present framework extends this work by specifying the institutional mechanisms, rather than the child's diagnosis, through which that burden is generated.

The researchers propose an Adapted Systemic Betrayal and Resilience Framework (see Figure 1) that integrates Smith & Freyd's (15) systemic betrayal with Ungar's (14) social-ecological model. The framework has four interdependent layers: Intellectual Disability as a Support Construct, understood through the AAIDD's definition in terms of the child's support needs and the interdependence and relational autonomy of the family unit, rather than as a deficit residing in the child (18); Systemic Integrity (versus Betrayal), whether institutions deliver accountable, compassionate care matched to those support needs; Caregiving Infrastructure, material resources like transport subsidies, grants, respite care, and therapy integration; and Relational and Meaning-Making Ecologies, family, peer networks, faith, and positive reframing that buffer psychological impact (19). These layers interact dynamically. Systemic integrity shapes infrastructure and relational ecologies, while systemic betrayal undermines both. Consistent with the supports-based definition of intellectual disability adopted here, the child with DS is not the cause of trauma; rather, parental trauma is a function of the mismatch between the child's support needs and the responses of surrounding systems. This reframing shifts the intervention from individual coping to institutional accountability.

**Figure 1:**
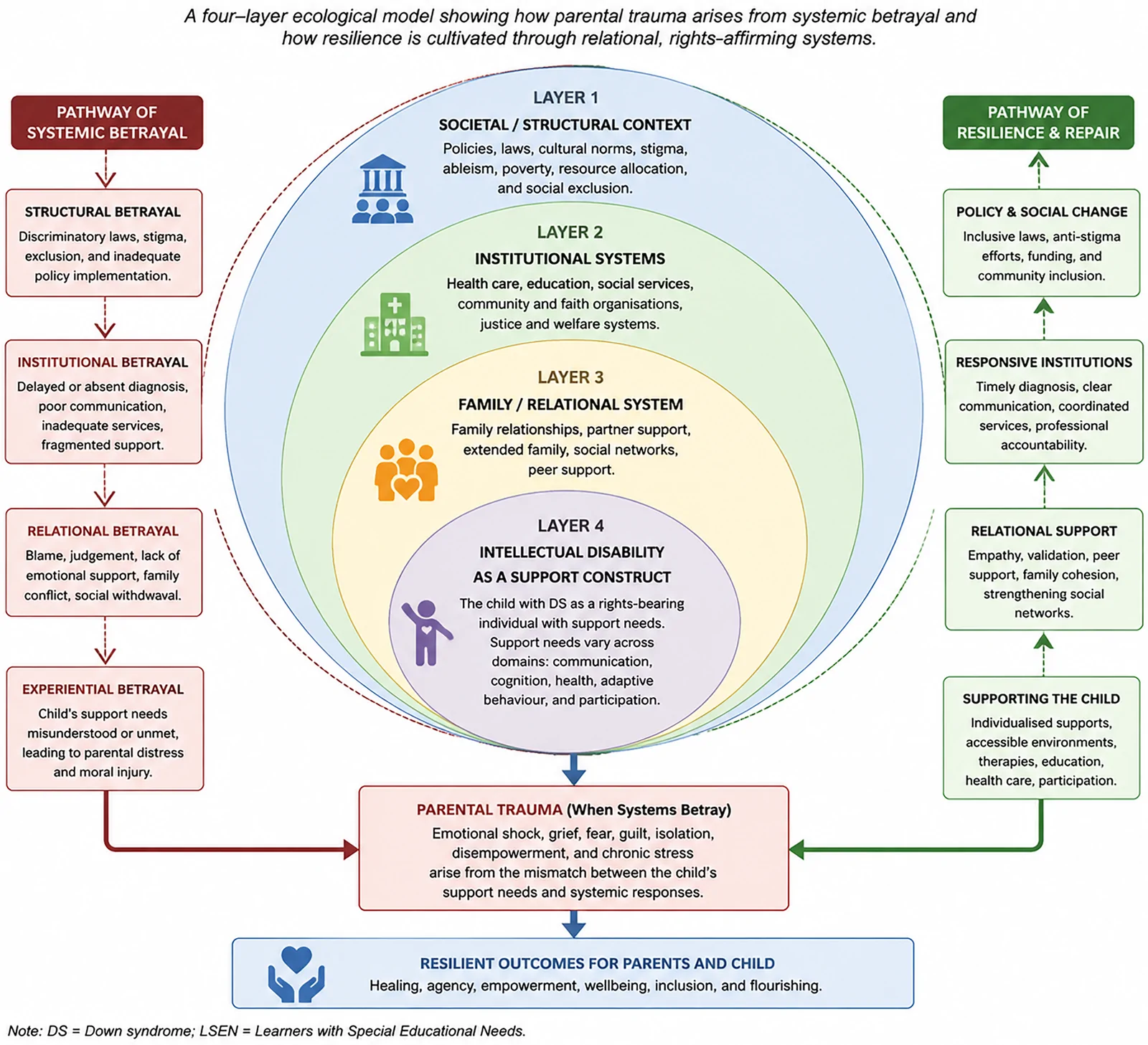
An adapted systemic betrayal and resilience framework for parental trauma in LSEN contexts.

### 2.1 Source: authors’ own work

This framework extends traditional applications of trauma theory by embedding individual psychological responses within a pragmatic, practice-oriented ecosystem. It provides a more realistic explanation of the dynamics at play in supporting parents of children with DS within LSEN contexts, moving beyond individualised coping models toward structural and institutional accountability. Recognition of trauma as an occupational hazard of disability caregiving under conditions of systemic betrayal reframes intervention: not only therapeutic support for parents but also, and more urgently, institutional reform to prevent trauma at its source.

## 3 Methodology

### 3.1 Research design

A qualitative design employing Interpretative Phenomenological Analysis (IPA) was adopted. IPA is particularly suited to research questions concerned with how individuals make sense of significant, often distressing, life experiences (20). The approach is idiographic, prioritising detailed engagement with each participant's account before cross-case analysis, and is explicitly interpretative, acknowledging the researcher's active role in meaning-making. The term parental trauma is used phenomenologically in this study to describe participants’ reported experiences of traumatic distress and systemic adversity; no clinical trauma or post-traumatic stress diagnosis was made.

### 3.2 Participants and setting

Eight parents (seven mothers, one father) of children with DS enrolled in a single LSEN school in Johannesburg South participated. Purposive sampling was used to select caregivers who were directly involved in their child's care and education. Participants ranged in age from 34 to 58 years and represented diverse ethnic backgrounds (Black African, Coloured, Indian). All were primary caregivers, and most reported significant financial constraints, with household incomes ranging from R500 to R8,000 monthly, primarily derived from social grants. At the average May–June 2025 exchange rate of approximately R18.00 to US$1 (ZAR/USD ≈ 0.0556 over the data-collection period), this corresponds to approximately US$28 to US$445 per month.

The LSEN school context is critical. The participating school provided on-site physiotherapy, speech therapy, occupational therapy, and a feeding scheme. It operated within Gauteng's under-resourced public education sector, with limited transport support and high staff workload.

The demographic profile of the eight participating parents is presented in Table 1.

**Table 1:** Demographic characteristics of participating parents (*N* = 8).

Participant	Gender	Age (years)	Relationship to Child	Household Income (ZAR)
P1	Male	46	Father	R5,000–R8,000
P2	Female	58	Mother	R500–R2,000
P3	Female	50	Mother	R2,000–R5,000
P4	Female	34	Mother	R500–R2,000
P5	Female	50	Mother	R500–R2,000
P6	Female	50	Mother	R2,000–R5,000
P7	Female	54	Mother	R500–R2,000
P8	Female	54	Mother	R2,000–R5,000

### 3.3 Data collection

Semi-structured individual interviews (30–50 min) and one focus group (60 min) were conducted between May and June 2025. Interviews explored parents’ experiences with diagnosis, schooling, navigating healthcare, community responses, coping strategies, and support systems. The semi-structured interview guide covered five domains: (i) the diagnostic experience and immediate aftermath; (ii) navigating healthcare and educational systems; (iii) everyday caregiving demands; (iv) community and interpersonal responses; and (v) coping resources and support networks. The focus group used a shorter, four-question guide addressing the same domains at a collective level, with the explicit purpose of eliciting socially embedded experiences (such as community stigma and peer coping) that are more readily articulated in a group setting than in one-on-one interviews. Written informed consent was obtained separately for the individual interviews and the focus group, using consent forms approved by the University of Johannesburg Faculty of Education Ethics Committee, which outlined the purpose of the study, the voluntary nature of participation, confidentiality arrangements, and participants’ right to withdraw. Questions were open-ended and designed to elicit narrative accounts rather than simple descriptions. All sessions were audio-recorded, transcribed verbatim, and anonymised using pseudonyms. All eight participants took part in both the individual interviews and the focus group.

While IPA is traditionally idiographic and case-centred, its application to focus group data has precedent in the literature (21, 22). The focus group in this study served a supplementary function: it was convened after individual interviews to provide a shared interactional space in which participants could articulate inherently social, contextually embedded experiences, such as community stigma and peer coping. This aligns with Love, Vetere and Davis’ (21) demonstration that IPA can be productively applied to group discourse, provided the analyst attends to both individual contributions within the group and the co-constructed meanings that emerge through interaction. Accordingly, focus group data were analysed following the same iterative IPA procedures applied to individual interviews, including initial noting, emergent theme development, and cross-case patterning. However, co-constructed utterances were treated with interpretive caution. Where a statement appeared to reflect group consensus or social desirability rather than individual meaning-making, this was noted in the reflexive memo and weighed accordingly in theme development. Individual interview data remained the primary analytic source; focus group data served to deepen, contextualise, and, where relevant, challenge emerging interpretations. The two data sources were crystallised rather than merely combined: individual-interview themes were developed first, and focus group material was then read against these themes to identify convergence, elaboration, or divergence, with any divergence noted explicitly in the analytic memos rather than resolved by default toward the individual-interview account. Saturation was assessed iteratively throughout the data collection process, with regular team discussions to confirm that sufficient depth had been achieved.

### 3.4 Data analysis

Data were analysed following IPA procedures: iterative reading, initial noting (descriptive, linguistic, conceptual), emergent theme development, and cross-case pattern identification. Reflexive memos were maintained throughout, and an audit trail documented analytic decisions. Member checking with three participants confirmed the resonance of preliminary themes. The first author (AHO) conducted the primary line-by-line analysis of all transcripts. To strengthen trustworthiness, peer debriefing was conducted throughout the analysis: the second author (VMD) independently reviewed a selection of initial notings and emergent themes against the raw transcript data, and interpretive discrepancies were resolved through discussion until consensus was reached; this discussion, rather than the individual coding pass alone, constituted the crystallisation step referred to above. This process supported the plausibility and coherence of the final superordinate themes, consistent with IPA's recognition that multiple valid interpretations may coexist (22). In addition, a thick contextual description of participants, the setting, and socio-structural conditions was maintained to support transferability, enabling readers to assess the relevance of the findings to comparable LSEN and disability caregiving contexts (23). Together with member checking conducted with three participants, these strategies collectively address credibility, dependability, and transferability as outlined by Yardley (23).

### 3.5 Ethical considerations

Ethical approval was obtained from the University of Johannesburg's Faculty of Education Ethics Committee (SEM 1-2025-098) and the Gauteng Department of Education. Informed consent was obtained, confidentiality was ensured through the use of pseudonyms and encrypted data storage, and participants were offered access to counselling support.

## 4 Results

These four superordinate themes captured parents’ experiences of caregiving for a child with DS within the low-resourced LSEN context studied here, as shaped by systemic and institutional conditions rather than by the child's disability itself: (1) traumatic shock at diagnosis and systemic betrayal; (2) cumulative caregiving strain as chronic trauma exposure; (3) secondary trauma from institutional gatekeeping and stigma; and (4) precarious resilience and post-traumatic growth.

### 4.1 Theme 1: traumatic shock at diagnosis and systemic betrayal

For most participants, the diagnostic encounter was not a neutral transfer of medical information but a profoundly disorienting and emotionally distressing event. Several mothers described learning of their child's DS diagnosis days or weeks after birth, sometimes unexpectedly and without adequate preparation or psychosocial support. Participant 8 described the delayed and disorienting nature of the diagnosis: “It was a shock…only when she was already six months old.” Another participant described the fear and uncertainty she experienced when the diagnosis was accompanied by additional health concerns that required further medical assessment and care: “It was not a nice feeling because of her condition and the other health concerns that emerged alongside it…I was panicking on myself…My main concern was…will she make it?” (Participant 3). The sense of systemic betrayal emerged clearly in accounts of differential treatment within healthcare settings. One mother contrasted her experience with that of other parents who had received referrals to specialised hospitals, suggesting discrimination based on racial categorisation: “I don't know, is it because I'm coloured, or what was the reason? All the other parents who gave birth to children with Down syndrome were referred to [a specialised referral hospital], except me” (Participant 7). These diagnostic and post-diagnostic experiences functioned as traumatic events not only because of the shock they induced but because they violated parents’ assumptions about institutional competence, fairness, and care (Table 3). The medical system, trusted to guide and protect, became a source of confusion, exclusion, and abandonment (24).

### 4.2 Theme 2: cumulative caregiving strain as chronic trauma exposure

Beyond discrete traumatic events, parents described the relentless, cumulative burden of caregiving as itself traumatising. This chronic exposure manifested as hypervigilance, meaning parents’ own sustained, self-initiated monitoring of the child's medical status (e.g., watching for signs of illness or medical complications), developmental progress, and exposure to social threats such as exclusion or mistreatment by peers, school staff, or community members; anticipatory grief (persistent worry about the child's future and the parent's mortality); and moral injury (distress arising from the inability to provide desired care due to resource constraints). Participant 7 captured the exhaustion of prolonged medical surveillance: “For the first two years of his life in hospital, my mother used to be there during the day, and at night, I slept in the hospital for two years until we decided to do the operation. It was very, very difficult.” Participant 6 described the fragmentation of medical care as a source of chronic disorientation and fatigue: “These kids … they've got too many appointments… It's like every part of the body, I have to go and make sure that it is functioning okay… You become tired in such a way that sometimes you have to skip work.” Anticipatory grief, the recurrent experience of loss before it occurs, surfaced in parents’ reflections on their own mortality and their children's uncertain futures: “I always wonder what will happen to my child when I die” (Participant 5, from the focus group). This pattern is consistent with the broader literature on chronic sorrow and anticipatory grief among parents of children with disabilities, including grief that resurfaces around unmet developmental milestones and persists independently of time since diagnosis (24, 25), and with qualitative evidence that ageing parents of individuals with intellectual and developmental disabilities carry longstanding, often unresolved worry about who will provide care after they themselves die or are no longer able to do so (26). The cumulative nature of these exposures suggests that parental trauma in LSEN contexts is not accurately conceptualised as a single-incident post-traumatic stress response but rather as a complex, layered condition arising from sustained caregiving under conditions of institutional precarity.

### 4.3 Theme 3: secondary trauma from institutional gatekeeping and stigma

Parents described encounters with educational institutions as sources of secondary trauma: that is, traumatic responses generated not by a direct threat to the physical safety of the parent or child, but by witnessing or experiencing systemic cruelty, exclusion, and humiliation directed at the family because of the child's disability. School admission experiences were particularly vivid in this regard. These admission experiences preceded and, in several cases, directly explain participants’ eventual enrolment of their children in the LSEN school at the centre of this study; the low-resourced LSEN context is therefore best understood not as a separate, later setting, but as the destination families reached after the exclusion described below. Participant 2 recounted a traumatic school rejection: “We went to a Catholic school, and there we couldn't even go through the gate. We went to [a second mainstream school]. As we went in to go and ask, a teacher came out. She said we haven't even asked anything, she said, ‘We don't take children like that.’ And that was the first time, and it was a shock to me, and I was very, very angry. I was so angry that I wanted to put it in the newspaper.” Participant 1 described a similar experience of being dismissed before any request could be made: “We haven't even asked anything… the teacher said, we don't take children like that when I wanted to enrol my child at a certain school.” Community stigma operated as another vector of secondary trauma.

Further, parents described the experience of having their child stared at, ignored, or actively avoided in public spaces; each encounter was a small but accumulating assault on dignity and belonging. Participant 5 articulated this painful dynamic: “When you walk with your child, then they will stare at her or at him like he's not a human being… some parents, when he goes there and greets them, sometimes they don't want to be greeted. Some… look at her as if she's disgusting”. These experiences of gatekeeping and stigma share features of betrayal trauma: they involve harm inflicted by institutions (schools) or communities that families depend upon for social integration and child development. The traumatic impact is amplified precisely because the rejecting school or neighbour is not a stranger but a member of one's moral community (Table 4).

### 4.4 Theme 4: precarious resilience and post-traumatic growth

Despite the traumatic exposures described, parents also articulated processes of adaptation, meaning-making, and, for some, post-traumatic growth (PTG), positive psychological changes arising from the struggle with adversity. These resilience processes, however, were described as precarious, dependent on unstable configurations of personal, relational, and institutional resources. Positive reframing emerged as a critical cognitive strategy. Parents actively worked to reinterpret the diagnosis not as a catastrophe but as an aspect of their child's unique identity. Participant 2 expressed this reframing through a lens of gratitude and faith: “I was overwhelmed with gratitude and thankfulness… I heard the diagnosis, but it did not disturb me. I was exceedingly grateful for the arrival of this diminutive purple infant.” It is worth noting that this account does not establish whether the participant had previously framed the diagnosis differently; it may reflect an initial response rather than a later reframing. Spiritual connection provided another anchor. Parents narrated faith not as denial but as a resource for sustaining meaning in the face of institutional and existential uncertainty. Participant 5 stated: “I believe that my child is a gift… When the Bible says God knows the plans He has for you, I tell myself He has plans for me as well. He's not a mistake.”

Relational support, particularly family solidarity and peer connections, functioned as critical buffers. Parents described siblings, grandparents, and extended kin stepping in to share caregiving tasks, providing both practical relief and emotional validation. Participant 4 noted: “My mother used to be there during the day at the hospital, and at night I slept there. Family is everything to me.” The LSEN school itself emerged as a paradoxical site: simultaneously, the place parents arrived at after prior trauma (rejection, stigma) elsewhere, where several described being able to speak about that earlier exclusion without fear of further rejection, and a source of current institutional support that mitigated ongoing strain. Parents valued the school's communication, integrated therapies, and sense of community. Participant 6 observed: “I've never seen a school that is communicating like this. Whatever they communicate, in every way they communicate, they make sure that you know.” However, resilience remained precarious. Financial strain, transport disruptions, institutional delays, and stigmatising encounters could rapidly erode adaptive coping. Parents described resilience as “taking one day at a time”, a stance that acknowledges the fragility of positive adaptation under conditions of chronic stress Table 2. Characteristics and illustrative quotes for superordinate themes of parental trauma.

**Table 2:** Characteristics and interpretive synthesis for superordinate themes of parental trauma.

Theme	Characteristic	Interpretive Synthesis (see main text for full quotations)
Traumatic Shock at Diagnosis and Systemic Betrayal	Diagnostic encounter as a disorienting, terrifying event; violation of assumptions about institutional competence and fairness.	Across accounts, the diagnostic encounter was recalled as abrupt and disorienting, often delivered without preparation, and, in one instance, compounded by an unexplained referral discrepancy that the participant read as differential treatment [Theme 1 above; cf. (15, 17)].
Cumulative Caregiving Strain as Chronic Trauma Exposure	The relentless, cumulative burden of caregiving manifests as hypervigilance, anticipatory grief, and moral injury.	Participants described caregiving as cumulative rather than episodic: prolonged hospital-based caregiving in infancy and an unrelenting schedule of appointments in later years both left parents physically and emotionally depleted [Theme 2 above; cf. (5, 27)].
Secondary Trauma from Institutional Gatekeeping and Stigma	Traumatic responses from experiencing systemic cruelty, exclusion, and humiliation by educational institutions and communities.	School-based rejection and public stigma were described as compounding sources of secondary trauma, ranging from formal exclusion at the point of enrolment to everyday experiences of being stared at or avoided [Theme 3 above; cf. (3, 4)].
Precarious Resilience and Post-Traumatic Growth	Adaptation, meaning-making, and positive psychological changes arising from adversity are dependent on unstable configurations of resources.	Positive reframing, spiritual meaning-making, and valued school communication each contributed to resilience, though participants’ accounts suggest these resources remained fragile and context-dependent rather than assured [Theme 4 above; cf. (28)].

**Table 3:** Trauma responses, coping strategies, and support systems of parents.

Component	Description and Key Manifestations	Interpretive Synthesis (see main text for full quotations)
Trauma Responses	**Hypervigilance:** Constant monitoring for medical, developmental, or social threats. **Anticipatory Grief:** Persistent worry about the child's future and the parent's mortality. **Moral Injury:** Distress from inability to provide desired care due to resource constraints.	Hypervigilance, anticipatory grief, and moral injury together formed a sustained rather than episodic burden, consistent with the broader literature on chronic sorrow and long-term care worry among parents of children and adults with intellectual and developmental disabilities [cf. (24–26)].
Cognitive and Spiritual Coping	**Positive Reframing:** Reinterpreting the diagnosis as part of the child's unique identity. **Spiritual Connection:** Faith as a resource for sustaining meaning without displacing biomedical care.	Faith and cognitive reframing operated together, allowing parents to hold the diagnosis as meaningful without displacing engagement with biomedical care [Theme 4 above; cf. (28)].
Relational Buffers	**Family Solidarity:** Siblings, grandparents, and extended kin sharing caregiving tasks. **Peer Connections:** Non-judgemental encouragement and rapid, practical information from other parents.	Extended family and peer networks functioned as practical, rapid-response buffers, supplying both hands-on caregiving support and time-sensitive information that formal services did not reliably provide (Theme 4 above).
Institutional Support (Paradoxical Site)	LSEN school is a location where prior trauma is processed and a source of current support; valued for communication, integrated therapies, and a sense of community.	Participants’ accounts of the LSEN school's communication practices were the most concrete evidence offered for this theme, illustrating what “valued for communication” meant in practice (Theme 4 above).

**Table 4:** Systemic barriers and support needs identified by parents.

Barrier/Need Domain	Nature of the Barrier/Need	Interpretive Synthesis (see main text for full quotations)
Diagnostic and Healthcare Pathways	Insensitive communication, fragmented appointments, lack of psychosocial support, and perceived discrimination in referral pathways.	Diagnostic communication and referral pathways were experienced as sources of acute distress in their own right, compounded in one account by a perceived racial disparity in referral practice, consistent with documented barriers to intellectual disability services in South African healthcare settings [cf. (26, 29)].
Institutional Gatekeeping and Educational Access	School rejection, stigmatisation by school staff, and exclusion from mainstream settings before any request could be made.	Exclusion from mainstream schooling occurred at the point of first contact, before any formal request or assessment had taken place, foreclosing rather than gatekeeping access [Theme 3 above; cf. (8, 9)].
Community Stigma	Public staring, avoidance, refusal of greetings, and treating the child as “disgusting” or “not a human being,” leading to accumulating assaults on dignity.	Community stigma was experienced as a cumulative, everyday assault on dignity rather than a series of discrete incidents, echoed in participants’ descriptions of public staring and refusal of greetings [Theme 3 above; cf. (3, 4)].

## 5 Discussion

This study examined parents’ experiences of trauma among caregivers of children with Down syndrome (DS) within LSEN school settings in Johannesburg, understood, consistent with the AAIDD-based conceptualisation of intellectual disability adopted in this paper, as arising from institutional and structural conditions rather than from the child's disability itself. The findings suggest that parental trauma is not best understood as a discrete reaction to diagnosis but rather as a layered and evolving process arising from the cumulative interaction of insensitive diagnostic communication, chronic caregiving demands, institutional barriers, and stigmatising social encounters. Participants described experiences characterised by hypervigilance, anticipatory grief, emotional exhaustion, and disruptions to previously held assumptions about family life and the future. These findings resonate with emerging evidence demonstrating that parents of children with intellectual and developmental disabilities (IDD) experience elevated levels of post-traumatic stress symptoms (PTSS) and trauma-related distress, often linked to ongoing caregiving challenges rather than isolated adverse events (17, 30, 31). Consistent with studies highlighting the chronic nature of disability-related caregiving stress (32, 33), the present findings suggest that trauma in this context is cumulative, relational, and embedded within everyday caregiving realities.

A particularly significant contribution of this study is the identification of systemic betrayal as a central mechanism through which trauma is produced and sustained. Participants depended on healthcare, educational, and social welfare institutions not merely as service providers but as essential infrastructures for their children's development and wellbeing. When these institutions failed through insensitive diagnostic communication, exclusionary school practices, fragmented service pathways, transport-related barriers, and inconsistent access to support, participants experienced these failures as profound violations of trust. This finding aligns closely with the growing literature on institutional betrayal, which demonstrates that harms perpetrated or enabled by trusted institutions often exacerbate psychological distress beyond the effects of the original stressor itself (34–37). Institutional betrayal has been associated with heightened distress, anger, distrust, service avoidance, and negative expectations of future support (37, 38). The mother's perception that referral pathways were applied differently to her is consistent with literature showing that healthcare access in South Africa remains stratified by race and class, with disadvantaged groups experiencing discrimination across the continuum of access, persistent racial disparities in healthcare use, and structural barriers within referral systems that hinder timely access to specialist care (29, 41). The persistent vigilance and mistrust reported by participants in the present study suggest that institutional failures may function not simply as administrative shortcomings but as trauma-generating experiences that undermine caregivers’ sense of safety and predictability.

The findings further indicate that parental trauma is deeply intertwined with structural and material conditions. Participants frequently described transport costs, fragmented appointments, inadequate financial assistance, and difficulties navigating complex service systems as persistent sources of stress (27). Participant 6, for example, described how a delay in grant renewal directly unravelled the family's transport and care arrangements: “The grant made it possible to keep transport paid… When the renewal was delayed, everything unravelled.” Such accounts support contemporary understandings of trauma as embedded within broader social-ecological contexts rather than residing solely within individuals (28, 39). While previous studies have identified barriers to care as significant predictors of parental PTSS (30, 31), the present study extends this literature by demonstrating how these barriers become incorporated into parents’ lived experiences of trauma, contributing to feelings of helplessness, exhaustion, and moral injury. The findings therefore challenge individualised approaches that conceptualise parental distress primarily as a deficit in coping and instead highlight the role of structural conditions in shaping psychological outcomes.

The LSEN school environment emerged as both a protective and constrained context. Participants identified school-based communication, therapeutic integration, and peer networks as important sources of support that mitigated some of the burdens associated with caregiving. Participant 6 illustrated this valued communication directly: “I've never seen a school that is communicating like this. Whatever they communicate, in every way they communicate, they make sure that you know.” Peer networks operated similarly: the same participant described how “within 10 min, five parents replied with exact questions to ask the clinic,” illustrating the practical, rapid support such networks provided. However, these supportive features did not eliminate exposure to trauma-generating experiences occurring across healthcare, welfare, and community systems. This finding supports social-ecological models of resilience, which conceptualise resilience as a multisystemic process arising from interactions among individual, relational, institutional, and structural resources (28, 33, 40). Consistent with this perspective, resilience in the present study was not located solely within parents themselves but emerged through access to supportive relationships, inclusive educational environments, faith communities, and opportunities for social participation. These findings suggest that interventions aimed at improving parental wellbeing should move beyond individual-level coping programmes and address broader systemic factors that contribute to chronic stress and trauma exposure.

Another important finding concerns the coexistence of trauma and post-traumatic growth (PTG). Participants reported experiences of strengthened relationships, deeper spirituality, enhanced appreciation of life, and the development of new forms of personal meaning, echoed, for example, in Participant 5's account of faith as a sustaining resource for reframing the diagnosis (see Theme 4). These findings mirror previous research demonstrating that PTG frequently coexists with significant psychological distress among parents of children with developmental disabilities (31, 32, 38). Rather than representing recovery from trauma, growth appeared to emerge through ongoing efforts to make sense of adversity and reconstruct meaning in the face of continuing challenges. Importantly, participants’ narratives caution against romanticising resilience or growth as evidence that institutional failures are inconsequential. Instead, meaning-making processes appeared to function as adaptive responses to structural adversity. As social-ecological approaches to resilience emphasise, individual strengths and relational resources can support adaptation but cannot substitute for equitable systems, accessible services, and institutional accountability (28, 40).

Taken together, the findings of this study support a conceptualisation of parental trauma among caregivers of children with DS as a multidimensional phenomenon shaped by the interaction of caregiving demands, institutional trustworthiness, structural conditions, and relational resources. The proposed ID-informed systemic betrayal and resilience framework advances existing scholarship by integrating trauma theory, institutional betrayal, social-ecological resilience perspectives, and a supports-based understanding of intellectual disability, to explain how trauma is both produced and mitigated across multiple levels of experience, without locating its cause in the child's disability. The findings underscore the need for trauma-informed diagnostic practices, integrated service delivery, accessible transport and financial support systems, and institutional cultures that actively foster trust, inclusion, and caregiver participation.

## 6 Conclusions

Among participants in this study, parental trauma experienced by caregivers of children with Down syndrome within LSEN school contexts was a complex and cumulative phenomenon that cannot be reduced to individual psychological vulnerability. Rather, it emerges through the intersection of insensitive diagnostic communication, chronic caregiving demands, institutional gatekeeping, community stigma, and broader structural inequalities. The findings indicated that trauma develops not from a single adverse event but through ongoing exposure to caregiving pressures, systemic barriers, and uncertainty, highlighting the need to recognise disability caregiving as a context in which trauma is socially and institutionally produced, with important implications for policy, practice, and future research.

A key finding is that systemic betrayal within healthcare, education, and social welfare systems can intensify parental distress by eroding trust and creating additional burdens. At the same time, parents drew on family relationships, peer support, faith communities, and school-based resources to foster resilience and, in some cases, experience post-traumatic growth despite continuing challenges.

The findings highlight the importance of understanding trauma and resilience as social-ecological processes influenced by both individual and systemic factors. They underscore the need for trauma-informed services, coordinated support systems, inclusive educational practices, and stronger social protection measures for families of children with Down syndrome in this and comparable low-resourced LSEN contexts. Ultimately, improving caregiver wellbeing requires not only strengthening parental resilience but also developing responsive and equitable institutions that reduce avoidable harm and promote family flourishing.

### 6.1 Limitations

Several limitations should be acknowledged. The sample was relatively small and drawn from a single LSEN school; consistent with the aims of qualitative inquiry, the intention is not statistical generalisability but a rich, context-specific understanding, and readers should judge the relevance of these findings to other LSEN or low-resourced settings based on similarity of context rather than assuming direct transferability. Fathers and other male caregivers were underrepresented, restricting understanding of gendered variations in trauma experiences. Furthermore, the cross-sectional design captured participants’ experiences at a single point in time and could not examine changes in trauma and resilience trajectories across the lifespan of caregiving. Future research would benefit from longitudinal, multisite studies that include more diverse caregiver groups and explore how institutional experiences shape parental wellbeing over time. This study is also unable to determine whether, and how, parents’ own ways of talking about their children's differences may at times echo the stigmatising attitudes they described encountering. Nor does the data directly capture children's own experiences of these encounters; the study relied on parents’ accounts of what they observed and inferred, not children's first-person perspectives. We also note, following the reviewer's caution, that framing a child's disability primarily as a source of meaning for the parent risks treating the child instrumentally rather than as a person of equal standing in their own right, a tension this study did not resolve and flags for future work.

### 6.2 Recommendations

The study recommends adopting trauma-informed approaches across education, health, and social systems, supporting children with DS and their families. In LSEN schools and health services, staff should be trained to recognise trauma, provide psychosocial support, offer compassionate communication of diagnoses, and establish referral and peer/parent support systems to reduce isolation.

Because these are qualitative findings from a single school, the following are offered as policy implications suggested by participants’ accounts rather than as tested policy conclusions. At the policy level, the Department of Basic Education should enforce inclusive admission policies and prevent unjust exclusion of children with DS, while linking funding to inclusive practices. The Department of Health should develop coordinated, integrated care pathways to reduce service fragmentation, and the South African Social Security Agency (SASSA) should adjust disability grants to account for inflation, reducing financial strain on families.

Future research should focus on long-term trauma outcomes, compare different school placement settings, and evaluate the effectiveness of trauma-informed interventions such as counselling, peer support, and family support services. Future research should also engage children with DS directly, using age- and communication-appropriate methods, to capture their own experiences of family life, schooling, and community belonging; the present study, having relied on parents’ accounts, was not able to represent children as active participants in their own right, and this recommendation follows from that gap rather than from evidence already presented above. Directly incorporating children's perspectives could also refine the proposed Adapted Systemic Betrayal and Resilience Framework, particularly Layer 4 (Intellectual Disability as a Support Construct), which, in its current form, characterises the child's support needs largely through parental report. Children's own accounts of exclusion, belonging, and institutional contact may converge with or diverge from parental accounts. Such divergence would itself be informative; it would indicate where parent-reported support needs are an incomplete proxy for the child's lived experience, pointing to a further layer, or a revision of Layer 4, in future applications of the framework.

## Data Availability

The original contributions presented in the study are included in the article/Supplementary Material, further inquiries can be directed to the corresponding author.
